# The role of genetics in neurodegenerative dementia: a large cohort study in South China

**DOI:** 10.1038/s41525-021-00235-3

**Published:** 2021-08-13

**Authors:** Bin Jiao, Hui Liu, Lina Guo, Xuewen Xiao, Xinxin Liao, Yafang Zhou, Ling Weng, Lu Zhou, Xin Wang, Yaling Jiang, Qijie Yang, Yuan Zhu, Lin Zhou, Weiwei Zhang, Junling Wang, Xinxiang Yan, Jinchen Li, Beisha Tang, Lu Shen

**Affiliations:** 1 Department of Neurology, Xiangya Hospital, Central South University, Changsha, China; 2 National Clinical Research Center for Geriatric Disorders, Central South University, Changsha, China; 3 Engineering Research Center of Hunan Province in Cognitive Impairment Disorders, Central South University, Changsha, China; 4 Hunan International Scientific and Technological Cooperation Base of Neurodegenerative and Neurogenetic Diseases, Changsha, China; 5 Key Laboratory of Hunan Province in Neurodegenerative Disorders, Central South University, Changsha, China; 6 Department of Geriatrics, Xiangya Hospital, Central South University, Changsha, China; 7 Key Laboratory of Organ Injury, Aging and Regenerative Medicine of Hunan Province, Changsha, China; 8 Department of Radiology, Xiangya Hospital, Central South University, Changsha, China

**Keywords:** Disease genetics, Alzheimer's disease, Disease genetics

## Abstract

Neurodegenerative dementias are a group of diseases with highly heterogeneous pathology and complicated etiology. There exist potential genetic component overlaps between different neurodegenerative dementias. Here, 1795 patients with neurodegenerative dementias from South China were enrolled, including 1592 with Alzheimer’s disease (AD), 110 with frontotemporal dementia (FTD), and 93 with dementia with Lewy bodies (DLB). Genes targeted sequencing analysis were performed. According to the American College of Medical Genetics (ACMG) guidelines, 39 pathogenic/likely pathogenic (P/LP) variants were identified in 47 unrelated patients in 14 different genes, including *PSEN1*, *PSEN2*, *APP*, *MAPT*, *GRN*, *CHCHD10*, *TBK1*, *VCP*, *HTRA1*, *OPTN*, *SQSTM1*, *SIGMAR1*, and abnormal repeat expansions in *C9orf72* and *HTT*. Overall, 33.3% (13/39) of the variants were novel, the identified P/LP variants were seen in 2.2% (35/1592) and 10.9% (12/110) of AD and FTD cases, respectively. The overall molecular diagnostic rate was 2.6%. Among them, *PSEN1* was the most frequently mutated gene (46.8%, 22/47), followed by *PSEN2* and *APP*. Additionally, the age at onset of patients with P/LP variants (51.4 years), ranging from 30 to 83 years, was ~10 years earlier than those without P/LP variants (*p* < 0.05). This study sheds insight into the genetic spectrum and clinical manifestations of neurodegenerative dementias in South China, further expands the existing repertoire of P/LP variants involved in known dementia-associated genes. It provides a new perspective for basic research on genetic pathogenesis and novel guiding for clinical practice of neurodegenerative dementia.

## Introduction

Neurodegenerative dementias are a group of clinically heterogeneous diseases with frequently overlapping symptoms, such as multi-cognitive impairments, behavioral changes, and movement deficits^1^. Alzheimer’s disease (AD) is the most common dementia worldwide, accounting for 60–80% of all dementia cases^2^. Frontotemporal dementia (FTD) is the second most common cause of neurodegenerative dementia after AD in patients younger than 65 years, responsible for 10.2% of cases^3^, and dementia with Lewy bodies (DLB) has been reported as being the second most common dementia subtype in older people following AD, accounting for 7.5% of all dementia cases^4^. However, the etiology of neurodegenerative dementias is still obscure, which is thought to be caused by a combination of ageing, environmental, and genetic factors.

Recently, substantial progress has been made regarding the molecular genetics of neurodegenerative dementias. *PSEN1*, *PSEN2*, and *APP* are recognized as three causative genes for familial AD (FAD), which explains the genetic background of 5–10% of early onset AD (EOAD, younger than 65 years). The estimated mutation frequencies of *PSEN1*, *APP*, and *PSEN2* in EOAD, are 80%, 15%, and 5%, respectively^5^. Likewise, FTD is a genetically and pathologically heterogeneous disorder with a higher incidence of familial cases than AD. Genetic etiology has been revealed in ~30–50% of FTD patients with a positive family history^6,7^. At present, more than 10 genes are related to FTD, and *MAPT*, *GRN*, and *C9orf72* are the most common, accounting for ~60% of all cases of inherited FTD^3^. In contrast, the genetic architecture of DLB remains largely elusive^8^. To date, only three genes have been confirmed to be related to DLB, including *APOE*, *GBA*, and *SNCA*. However, growing evidence supports that DLB has a strong and unique genetic component^9^.

Interestingly, previous studies have suggested a potential genetic overlap between AD, FTD, and DLB. Notably, *PSEN1*, the most common etiology of EOAD, has also been found in patients with FTD and DLB^10–13^. Similarly, mutations in *MAPT*, *GRN*, and *C9orf72* have also been detected at lower frequencies in AD and DLB patients^14–16^. Homozygosity for *APOE4*, the strongest genetic risk factor for AD, has also been reported in several studies to increase the risk of FTD and DLB^17,18^. In addition, mutations in *SNCA* have been shown to result in a wide phenotypic spectrum of DLB, Parkinson’s disease (PD), multiple system atrophy (MSA), and FTD^19–21^.

In this study, we comprehensively analyzed the mutational spectrum of known dementia-associated genes from patients with neurodegenerative dementias in the South Chinese population using integrated targeted gene sequencing analysis. First, we systematically identified pathogenic and likely pathogenic (P/LP) variants of known dementia-associated genes, including known and novel variants, summarized and compared the mutation frequency among patients with different clinical diagnosis. Second, we generalized the clinical manifestation of neurodegenerative dementia patients carried P/LP variants in this study, including *PSEN1*, *PSEN2*, *APP*, *MAPT*, *GRN*, *C9orf72*, *CHCHD10*, *HTRA1*, *TBK1*, *OPTN*, *SQSTM1*, *VCP*, *SIGMAR1*, and *HTT*, attempting to summarize the relationship between gene mutations and clinical phenotypes. Then, we compared the age at onset (AAO) of patients with and without P/LP variants and patients carried different genes separately, to depict the AAO spectrum for these dementia-associated genes in our population. Finally, we analyzed *APOE* genotypes (non-carriers or carriers of *APOE4*) in AD cohort and conclude the difference between *APOE* genotypes and different AD subgroups. Our studies provide a new perspective for further basic research of neurodegenerative dementia, especially genetic-associated pathogenesis and facilitated the clinical prediction, diagnosis, and genetic counseling.

## Results

### Demographics and analysis of genes targeted sequence

**Fig. 1:**
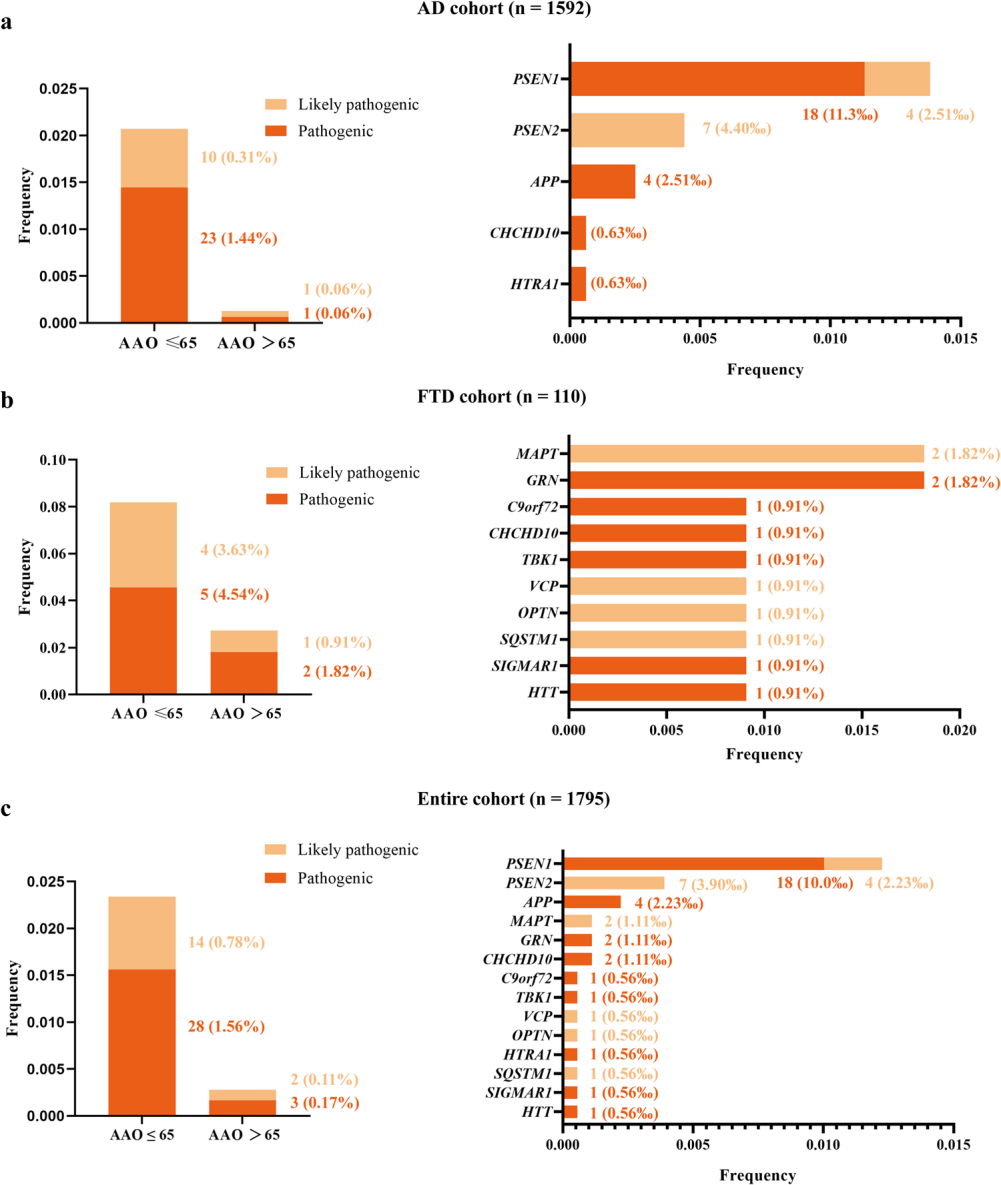
Mutational frequencies of known cognitive impairment disease-associated genes in AD, FTD, and entire cohorts respectively. Mutational frequencies of all (left) and each (right) known cognitive impairment-associated genes in the different dementia cohorts. **a** AD cohort. **b** FTD cohort. **c** entire cohort. Variants that were classified as pathogenic or likely pathogenic according to the standards and guidelines of the ACMG. ‘Pathogenic’ means that the patients had pathogenic variants in known cognitive impairment disease-associated genes, and ‘likely pathogenic’ means that the patients had likely pathogenic variants in known cognitive impairment disease-associated genes. ACMG American College of Medical Genetics, AD Alzheimer’s disease, FTD frontotemporal dementia.

In this study, 1592 AD patients, 110 FTD patients, and 93 DLB patients were included. Demographic and clinical characteristics are shown in Table 1. A total of 39 P/LP variants from 14 genes are identified in 47 unrelated patients by a dementia-related gene panel, which contained 36 genes associated with cognitive impairment phenotype (Supplementary Table 1 and Supplementary 2). Among them, 33.3% of variants (13/39) were novel, including *PSEN1* (c.451G>A, c679A>c, c.A1139>G, and c.1369 A>G), *PSEN2* (c.T716C and c.1180delG), *GRN* (c.20G>A), *CHCHD10* (c.121C>T and c.283C>T), *OPTN* (c.1402_1407del), *SQSTM1* (c.558_559insC), *SIGMAR1* (c.26G>A), and *TBK1* (c.973dup). All identified P/LP variants were responsible for 2.2% of AD (35/1592) and 10.9% of FTD (12/110), which led to an overall molecular diagnostic yield of 2.6% (Fig. 1), however, in this study, no P/LP variants were identified in DLB patients. 70.2% (33/47) of patients had a positive family history and 46.8% (22/47) of patients with P/LP variants had at least one *APOE4*.

**Table 1:** Summary of clinical features of the cognitive impairment disease patients in this study.

Clinical features	AD (*n* = 1592)	FTD (*n* = 110)	DLB (*n* = 93)	Total (*n* = 1795)
Age at onset, years	64.7 ± 10.8	59.6 ± 11.5	65.2 ± 9.9	64.5 ± 10.9
Gender (M, %)	645, 40.4%	56, 50.9%	57, 61.3%	758, 42.1%
Age at diagnosis, years	67.7 ± 10.9	62.4 ± 11.8	68.0 ± 9.6	67.4 ± 11.0
Family history (+, %)	467, 29.3%	35, 31.8%	16, 17.2%	518, 28.8%
Disease duration, years	3.0 ± 2.4	2.8 ± 2.3	2.9 ± 2.8	3.0 ± 2.4
Education attainment, years	8.5 ± 4.1	9.0 ± 4.0	7.7 ± 4.0	8.5 ± 4.1
MMSE	13.8 ± 8.5	15.3 ± 9.2	15.7 ± 7.7	14.0 ± 8.5
*APOE* genotypes (*n*, %)				
* APOE*4 +/+	145, 9.1%	5, 4.5%	7, 7.5%	157, 8.7%
* APOE*4 +/−	561, 35.2%	36, 32.7%	29, 31.2%	626, 34.9%
* APOE*4 −/−	886, 55.7%	69, 62.8%	57, 61.3%	1012, 56.4%

The age at onset, the age at diagnosis, disease duration, educational attainment, MMSE scores are all shown as mean ± standard deviation. Gender, family history, *APOE* genotypes are all shown as numbers and proportions (%).

*AD* Alzheimer’s disease, *FTD* frontotemporal dementia, *DLB* dementia with Lewy bodies, *MMSE* Mini-Mental State Examination.

### Mutational spectrum of the AD cohort

Overall, 27 different P/LP variants were identified in 35 unrelated AD patients from five genes, including *PSEN1*, *PSEN2*, *APP*, *CHCHD10*, and *HTRA1* (Table 2). 88.6% (31/35) carrier were FAD probands, 11.4% (4/35) were sporadic AD (SAD) cases.

**Table 2:** Clinical characteristics and variants information of patients with pathogenic or likely pathogenic mutations.

Genes	Base change	Protein change	No. of cases	Reported and references	Gender	AAO (ys)	*APOE* genotype	Family history	Clinical phenotypes				Clinical diagnosis
									Memory decline	Language impairment	Mental and behavior change	Sensory and movement disorders	
*PSEN1*	c.250A>G	p.M84V	1	Y^63^	M	53	3/4	+	+	+	+	+	AD
	c.415A>G	p.M139V	1	Y^64^	M	53	2/3	+	+	+	+	−	AD
	c.415A>T	p.M139L	1	Y^65^	F	38	4/4	+	+	−	−	+	AD
	c.424G>A	p.V142I	2	Y^66^	M/M	54/52	3/3	+/+	+/+	+/−	−/+	−/−	AD
	c.436A>G	p.M146V	1	Y^64^	M	42	3/4	+	+	+	+	−	AD
	c.451G>A	p.V151M	1	N	M	49	3/4	+	+	+	+	−	AD
	c.519G>T	p.L173F	1	Y^67^	F	37	3/4	+	+	−	+	−	AD
	c.604A>T	p.I202F	1	Y^68^	F	46	3/4	+	+	+	−	−	AD
	c.617G>A	p.G206D	1	Y^69^	M	38	3/3	+	+	−	+	−	AD
	c.677T>G	p.L226R	1	Y^70^	M	44	3/4	+	+	−	+	−	AD
	c.679A>C	p.I227L	1	N	M	44	3/4	−	+	−	−	−	AD
	c.697A>G	p.M233V	1	Y^71^	M	30	3/3	+	+	−	−	+	AD
	c.791C>T	p. P264L	2	Y^72^	M/F	51/52	3/4	+/−	+/+	+/+	+/−	+/−	AD
	c.806G>A	p.R269H	2	Y^73^	M/F	45/60	3/4	+/+	+/+	+/−	−/−	−/−	AD
	c.845T>G	p.L282R	1	Y^74^	F	52	3/3	+	+	+	−	+	AD
	c.854C>T	p.A285V	1	Y^75^	F	46	3/3	+	+	+	−	−	AD
	c.1139A>G	p.K380R	1	N	F	49	3/4	+	+	−	−	−	AD
	c.1174C>G	p.L392V	1	Y^76^	F	46	3/3	+	+	−	+	−	AD
	c.1369A>G	p.M457V	1	N	M	66	3/4	+	+	−	+	−	AD
*PSEN2*	c.715A>G	p.M239V	4	Y^77^	M/M/F/F	47/60/53/45	50% 3/3 50% 3/4	+/+/+/+	+/+/+/+	−/−/+/−	−/+/+/+	−/−/−/+	AD
	c.716T>C	p.M239T	1	N	F	50	3/4	−	+	−	−	−	AD
	c.717G>A	p.M239I	1	Y^32^	F	50	3/4	+	+	−	+	−	AD
	c.1180delG	p.A394Pfs*8	1	N	F	68	3/3	+	+	−	+	−	AD
*APP*	c.2143G>A	p.V715M	1	Y^78^	F	51	3/3	+	+	−	+	−	AD
	c.2149G>A	p.V717I	3	Y^79^	F/F/M	47/49/42	66.7% 3/3; 33.3% 3/4	+/+/+	+/+/+	+/+/+	−/+/+	−/−/−	AD
*MAPT*	c.1788T>G	p.N596K	1	Y^80^	F	42	3/3	−	+	−	−	+	FTD
	c.1907C>T	p.P636L	1	Y^81^	M	58	3/3	−	+	+	+	−	FTD
*GRN*	c.20G>A	p.W7*	1	N	F	73	3/4	+	+	−	+	−	FTD
	c.328C>T	p.R110*	1	Y^82^	F	61	3/3	−	+	+	+	−	FTD
*CHCHD10*	c.283C>T	p.Q95*	1	N	F	52	3/4	+	+	+	+	+	AD
	c.121C>T	p.Q41*	1	N	F	56	3/4	−	+	−	+	−	FTD
*HTRA1*	c.589C>T	p.R197*	1	Y^83^	F	49	3/3	−	+	−	+	−	AD
*OPTN*	c.1402_1407del	p.468_469del	1	N	F	63	3/3	−	+	+	−	−	FTD
*SQSTM1*	c.558_559insC	p.V287Rfs*21	1	N	F	71	3/3	−	+	+	−	−	FTD
*VCP*	c.475C>T	p.R159C	1	Y^84^	M	51	3/3	−	+	−	+	−	FTD
*SIGMAR1*	c.26G>A	p.W9*	1	N	F	74	3/3	−	+	−	+	−	FTD
*TBK1*	c.973dup	p.Y325Lfs*4	1	N	M	61	3/3	−	+	+	+	−	FTD
*C9orf72*	Hexanucleotide expansion	−	1	Y	F	57	3/4	+	+	+	−	+	FTD
*HTT*	CAG repeat expansion	–	1	Y	F	43	3/3	+	+	+	−	+	HD

*Y* mutations have been reported previously, *N* mutations have not been reported previously.

**Fig. 2:**
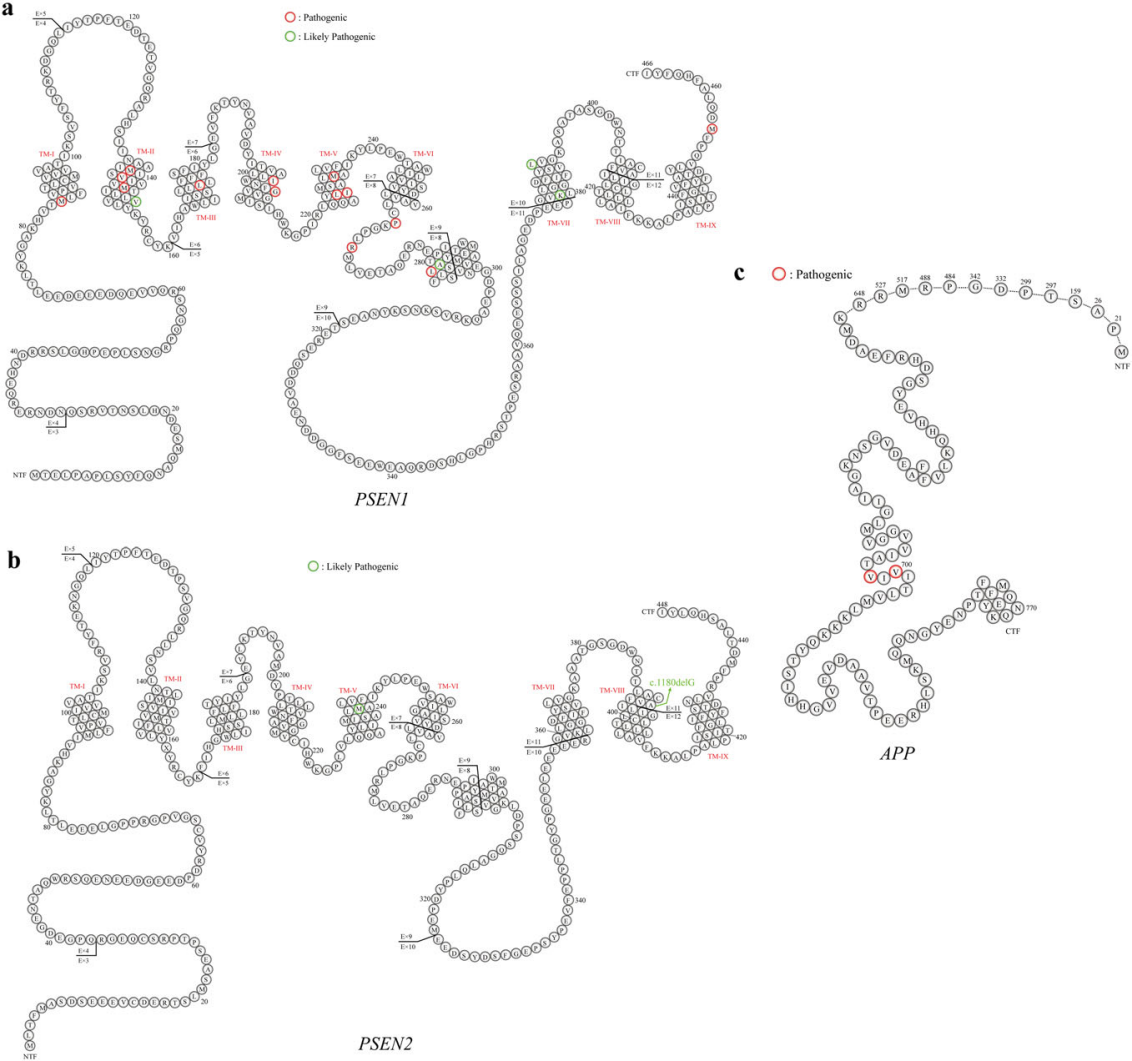
Distribution of amino acid substitutions in the PSEN1, PSEN2, and APP proteins. Red circles: pathogenic mutations identified. Green circles: likely pathogenic mutations identified. A Ala, C Cys, D Asp, E Glu, F Phe, G Gly, H His, I Ile, K Lys, L Leu, M Met, N Asn, P Pro, Q Gln, R Arg, S Ser, T Thr, V Val, W Trp, Y Tyr, APP amyloid precursor protein, PSEN presenilin. **a** The distribution of PSEN1 P/LP variants. **b** The distribution of PSEN2 P/LP variants. **c** The distribution of APP P/LP variatns.

In this study, *PSEN1* was the most frequently mutated gene, 19 P/LP missense mutations were identified in 22 patients, among which four were novel identified in our study, including c.451G>A, p.V151M; c.679A>C, p.I227L; c.1139A>G, p.K380R; and c.1369A>G, p.M457V. Seven patients carried *PSEN2* P/LP variants, including six missense mutations at the same amino acid residue (M239) and one frameshift mutation. Two were novel, including c.T716C, p.239M>T and c.1180delG, p.A394Pfs*8. All patients with the variants had a positive family history except for one who carried *PSEN2* p.239M>T. Meanwhile, two *APP* missense mutations were identified in four FAD probands, including c.2143G>A, p.V715M, and c.2149G>A, p.V717I (Table 2). The distribution of *PSEN1/PSEN2/APP* P/LP variants are shown in Fig. 2. Interestingly, all identified P/LP variants of *PSEN1*/*PSEN2*/*APP* were located in hydrophobic regions or in the endoproteolytic cleavage regions.

In patients with *PSEN1/PSEN2/APP* variants, 93.9% (31/33) were defined as early-onset AD (EOAD) (AAO < 65 years)), 45.5% were *APOE4*-negative, 48.5% had one *APOE4*, and 6.0% had two copies of *APOE4*.

About clinical phenotypes, all *PSEN1/PSEN2/APP* variants carriers initially presented with memory decline. Then, language impairment and behavior change were common symptoms in these variants, 52.63% (10/19) *PSEN1*, 25% (1/4) *PSEN2*, and 50% (1/2) *APP* P/LP variants showed language impairment, respectively, such as naming difficulty, repetitive speech, fluency disorder, and speech reduction, while the frequency of mental and behavior change of *PSEN1, PSEN2, APP* variants were 57.6% (11/19), 75% (3/4), and 100% (2/2), respectively. Meanwhile, 26.3% (5/19) *PSEN1*, 25% (1/4) *PSEN2* P/LP variants presented sensory and movement disorders, such as hallucination, delusion, weakness, involuntary movement, and abnormal gait. Interestingly, the clinical manifestation of patients with *PSEN2* mutations at amino acid residue M239 showed high heterogeneity, including memory decline, language impairment, mental and behavior change, and sensory and movement disorders.

Additionally, we also found two female AD patients who carried a nonsense mutation in *CHCHD10* (c.283C>T, p.Q95*) and *HTRA1* (c.589C>T, p.R197*), respectively. The patient who carried *CHCHD10* p.Q95* showed memory decline at 52 years and gradually developed language dysfunction, behavioral changes, bradykinesia, and depression. Brain MRI showed bilateral atrophy of temporal parietal lobe and hippocampus, and cerebrospinal fluid (CSF) examination showed the level of Aβ42 and Aβ42/Aβ40 ratio decreased, while the phospho-tau (p-tau) and total tau (t-tau) increased. In addition, the Pittsburgh compound B (PiB)-PET showed diffuse amyloid deposition in the whole brain cortex. The patient who carried *HTRA1*, p.R197*, mainly presented typical forgetfulness of recent events and daily living ability declined at 49 years. Brain MRI showed multiple spot-like hyperintensities in the deep bilateral frontotemporal lobes and paraventricular region, while no microbleeds on susceptibility-weighted images sequence. The level of Aβ42 in CSF decreased, and p-tau increased which supported the diagnosis of AD.

### Mutational spectrum of the FTD cohort

A total of 12 P/LP variants in 10 genes were identified in the FTD cohort, including *MAPT*, *GRN*, *C9orf72*, *CHCHD10*, *TBK1*, *OPTN*, *SQSTM1*, *VCP*, *SIGMAR1*, and *HTT*, summarized in Table 2. Six were novel variants, including three nonsense mutations (*GRN:* c. 20G>A, p.W7*; *CHCHD10:* c. 121C>T, p.Q41*; and *SIGMAR1*: c.26G>A, p.W9*), two frameshift mutations (*TBK1*: c.973dup, p.Y325Lfs*4 and *SQSTM1*: c.558_559insC, p.V287Rfs*21), and one deletion mutation (*OPTN*: c.1402_1407del, p.468_469del). Interestingly, only two P/LP variants carriers had a positive family history. The mean ± SD AAO of P/LP variants carriers was 59.2 ± 10.5 years, which was significantly older than P/LP variants carriers in AD cohort (48.8 ± 7.7, *p* = 0.001). Meanwhile, only three patients carried *APOE4* (25%, 3/12), which tended to be lower than P/LP variant carriers in the AD cohort (54.3%, *p* = 0.077).

As for clinical characteristics, all patients carried P/LP variants in FTD cohort showed memory decline, 58.33% (7/12) patients had language impairment, mental, and behavior changes, and 25% (4/12) P/LP variants carriers accompanied by sensory and movement disorders. Interestingly, one showed personality changes and language impairment, as well as abnormal emotional responses at baseline. In the fifth year of onset, she suffered from memory decline. Brain MRI showed bilateral frontal lobe atrophy, and bvFTD was initially considered. However, molecular testing revealed that she carried heterozygous CAG expanded repeats in *HTT*, which supported the diagnosis of Huntington’s disease (HD).

### Spectrum of age at onset

**Fig. 3:**
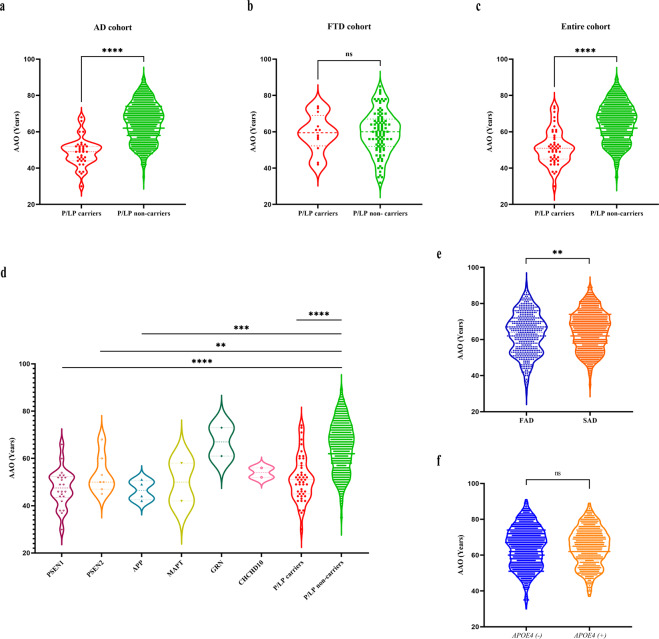
AAO spectrum of known cognitive impairment disease-associated genes in AD, FTD, and entire cohorts. Comparison of AAO in all patients with P/LP variants of known cognitive impairment disease-associated genes and patients without P/LP variants in known cognitive impairment disease-associated genes. **a** AD cohort. **b** FTD cohort. **c** Entire cohort. The dashed red line refers to the mean AAO of patients with P/LP variants in the corresponding cohorts, whereas the dashed line refers to the mean AAO of patients without P/LP variants in known cognitive impairment disease-associated genes in the corresponding cohort. **d** Spectrum of AAO in patients with P/LP variants of each cognitive impairment disease-associated gene (only genes carried by two or more patients were included), in patients with and without P/LP variants of known cognitive impairment disease-associated genes. **e** Spectrum of AAO in FAD and SAD patients. **f** Spectrum of AAO in patients with and without *APOE4*. *p < 0.05, **p < 0.01, ***p < 0.001, ****p < 0.0001, ns no significance. AAO age at onset, AD Alzheimer’s disease, FTD frontotemporal dementia, P/LP pathogenic or likely pathogenic, FAD familial Alzheimer’s disease, SAD sporadic Alzheimer’s disease.

Moreover, the mean AAO were significantly younger in patients with P/LP variants in the AD cohort and entire cohort, while no difference was found in the FTD cohort (Fig. 3a–c). Specifically, the mean AAO of patients with P/LP variants in the entire cohort was 51.4 ± 9.5 years, ~10 years younger than the mean AAO of those without P/LP variants (64.8 ± 10.7 years) (*p* < 0.001), among them, 89.4% were younger than 65 years.

Meanwhile, we analyzed the spectrum of AAO in patients with P/LP variants of different genes (genes with two or more mutations were included). The results showed that the mean AAO of subjects with P/LP variants of *PSEN1*, *PSEN2*, and *APP* (47.5 ± 10.7 years, 53.2 ± 8.1 years, and 46.5 ± 4.2 years, respectively) were significantly lower than those of non-carriers (*p* < 0.05) (Fig. 3d).

In addition, we performed subgroup analysis on the family history and the status of *APOE4* to compare the difference in AAO between the two groups respectively, which showed that the AAO of FAD patients was significantly younger than that of SAD (63.2 ± 11.4 and 64.9 ± 10.6, respectively, *p* = 0.005), while no significant between *APOE4* carriers and non-carriers (*p* = 0.953) (Fig. 3e, f).

To analyze the confounding factors affecting AAO, we conducted further multiple linear regression analysis with AAO as the dependent variable. After controlling independent variables, including gender, disease duration, educational attainment, *APOE* genotypes, dementia family history, MMSE scores, mutation status, and clinical diagnosis, the model showed that MMSE scores (*B* = −0.135, *p* < 0.001), disease duration (*B* = −0.421, *p* = 0.001), and status of mutation carried (*B* = −13.44, *p* < 0.001).

### Characteristics *of APOE* genotypes

**Fig. 4:**
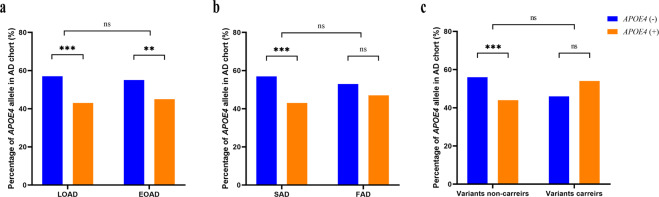
The percentage of *APOE4* in AD cohort. **a** The percentage of *APOE4* in LOAD and EOAD. **b** The percentage of *APOE4* in SAD and FAD. **c** The percentage of *APOE4* in AD patients with and without P/LP variants. *p < 0.05, **p < 0.01, ***p < 0.001, ****p < 0.0001, ns no significance. LOAD late-onset Alzheimer’s disease, EOAD early-onset Alzheimer’s disease, SAD sporadic Alzheimer’s disease, FAD familial Alzheimer’s disease.

As for the distributions of *APOE* genotypes, there was no significant difference across AD, FTD, and DLB cohorts (*p* > 0.0166; Bonferroni corrected). Furthermore, *APOE4 as* the strongest genetic risk factor for AD, we further compared the distribution difference between EOAD and LOAD patients, FAD and SAD. We found no significant difference in *APOE4* frequency (EOAD vs LOAD: *p* = 0.501; FAD vs SAD: *p* = 0.153, respectively). Further subgroup analysis in AD cohort showed that the proportion of *APOE4*-negative patients was higher than that of *APOE4*-positive patients (*p* < 0.001, *p* = 0.007, and *p* < 0.001, respectively) (Fig. 4a, b). In addition, we found no significant difference in the distribution difference of *APOE4* between variants carriers and non-carriers in the AD cohort (*p* = 0.281). Meanwhile, a higher percentage of *APOE4* (+) patients was found in P/LP variants than in patients without P/LP variant in AD cohort (*p* < 0.001) (Fig. 4c).

## Discussion

In this study, we determined the mutational spectrum of 36 known dementia-associated genes in patients clinically diagnosed with neurodegenerative dementia patients, including AD, FTD, and DLB in a South China population sample using integrated targeted gene sequencing analysis. This is the first report of distributions of gene mutations in patients with neurodegenerative dementias from South China. We observed that the use of an integrated gene analysis could be an effective tool for detecting potential genetic causes in neurodegenerative dementias with high genetic heterogeneity or overlapping phenotypic features, gaining further insight in genetic pathogenesis of neurodegenerative dementia, clinical diagnosis, and genetic counseling.

Mutations in *PSEN1* are the most common cause of EOAD, meanwhile, *PSEN1* was the most frequently mutated gene in patients with FAD. To date, more than 300 mutations in *PSEN1* have been identified to be associated with FAD. In this study, four novel variants were identified, which expanded the mutational spectrum of *PSEN1*. The AAO of *PSEN1* mutation carriers in our study (47.5 years), was older than Ryan et al. reported (43.6 years) in 168 AD patients with *PSEN1* mutations^22^, but younger than Jia et al. reported in a large FAD cohort from China (50.59 years)^23^. Of interest, in addition to the *PSEN1* mutations mentioned above, we found an older female (83 years) carrying a novel mutation (M270L), and the *APOE* genotype was 3/4 in the DLB cohort. Several algorithms predicted the variant was not disease damaging, whereas the nearby mutations (R269G, R269H, and L271V) have been reported to be associated with FAD^24–26^. Whether the clinical phenotype of the patient is caused by the novel mutation or the contribution of *APOE* genotype is unclear; we will perform functional research to further clarify the variant. Meanwhile, regarding clinical phenotypes, *PSEN1* mutation carriers often present with atypical cognitive symptoms and additional neurological features^22^. However, in this study, patients mainly presented with amnesia, language impairment, mental and behavioral changes, and movement disorders, which is one limitation of this study. This might have two explanations. First, some atypical symptoms might not occur at an early stage of the disease, and follow-up is necessary. Second, the mutation locations may lead to distinguishing phenotypes; for example, atypical cognitive presentations and pyramidal signs were seen more frequently in association with *PSEN1* mutations involving exon 8^22^, suggesting that multiple factors could contribute to the phenotypic heterogeneity of *PSEN1*-related AD.

In contrast to *PSEN1*, only 18 pathogenic mutations within *PSEN2* have been reported, most of which occurred in European and African populations. In this study, seven P/LP variants were identified, including six FAD cases. Previous studies showed that the AAO of *PSEN2*-associated cases vary widely, from 45 to 88 years; that is more than 10 years later than the mean AAO for *PSEN1*-related cases^27–29^, which was consistent with our results. Interestingly, six patients with a substitution at *PSEN2* amino acid residue 239 were identified, including M239V, M239I, and M239T. Among them, M239V has been reported in European populations to elevate Aβ42 levels and Aβ42/Aβ40, and to exhibit a partial loss of function with respect to the C-terminal fragment-γ as well as a substantial decrease in Aβ40 levels^30–32^, but it has been absent from Asian cohorts so far. Our findings suggest that this residue may be a common causative variant in the South Chinese population. In addition, the clinical phenotypes of carriers of the M239V mutation varied widely. Our findings, together with previous reports, further suggest that phenotypic heterogeneity exists even at the same codon site because of different amino acid transversions^30,32,33^.

In accordance with other populations, the common mutation site of *APP* were residues 715 and 717. Taken together with our previous reports, our team have found four families carrying mutations at this site^34^. Amino acid residues 715 and 717 are located near the γ-secretase cleavage site, and mutation at this site may increase the hydrophobicity of the *APP* TM domain to anchor the protein within the membrane and elevate the Aβ42/Aβ40 ratio^35,36^. Interestingly, patients with mutations at this site often have non-memory symptoms, which can be misdiagnosed as FTD, because the behavioral problems occur earlier than the memory deficits. Totally, in all *PSEN1*/*PSEN2*/*APP* P/LP variants in LOAD, *PSEN1* M457V, and *PSEN2* A394Pfs*8 were novel variants, further functional validation was necessary and warranted.

In addition, we identified *CHCHD10* and *HTRA1* mutations in the AD cohort. *CHCHD10* has been identified to be associated with a large spectrum of diseases, including FTD, ALS, AD, cerebellar ataxia, mitochondrial myopathy, late-onset spinal motor neuronopathy, and Charcot-Marie-Tooth disease type 2^37–40^. Previously, we have reported a late-onset AD patient with the *CHCHD10* mutation^41^. A homozygous *HTRA1* mutation was known to be causative for CARASIL^42^, while evidence was also showed that heterozygous *HTRA1* mutation, which might result in an impaired *HTRA1* activation cascade or be unable to form stable trimers, is related to autosomal dominant hereditary cerebral small vessel disease with delayed onset^43–45^. In this study, the female AD patients presented with typical amnesia symptoms at 49 years without any other neurological or extra-neurological symptoms. Meanwhile, the Fazekas score of periventricular white matter hyperintensities was 1, the *APOE* genotype was 3/3 and the core biomarkers of CSF showed A+T+N-. However, the effect of the heterozygous mutation in the pathophysiologic process of AD remains elusive; further functional studies are still needed. These results further indicate that mutations not only in *PSEN1*, *PSEN2*, and *APP* can cause the AD phenotype, but that variants in other genes might also cause AD-like symptoms. Further follow-up is necessary.

Notably, we identified double mutations in a 52-year-old female (*PSEN2* p.M239I and *MAPT* p.R5H), but her daily living ability remained intact, and the double mutation did not accelerate the cognitive decline, further expanding the phenotype spectrum of the mutation and supporting the phenotypic heterogeneity among subjects carrying the same *MAPT* mutations. Further in vivo and in vitro studies are needed to determine the effect of *MAPT* and *PSEN2* mutations on the pathology and pathogenesis of AD.

Many different gene are reported to cause FTD, of which *MAPT*, *GRN*, and *C9orf72* are three most common^46–48^. Except of three common genes, we also found variants in another seven genes, including *CHCHD10*, *OPTN*, *SQSTM1*, *VCP*, *SIGMAR1, TBK1*, and *HTT*^49–52^. In addition to genetics, the clinical phenotypes of FTD are also highly heterogeneous. In this study, we did not observe the classic phenotypes of mutations in *VCP*, such as inclusion body myopathy with Paget’s disease of the bone^53,54^, we will follow up the patient to see the symptoms evolve. Moreover, the wrong diagnosis of the patient carried heterozygous CAG expanded repeats in *HTT*, further indicated that the overlap of clinical phenotypes is one of the main reasons for the difficulty in the diagnosis of neurodegenerative diseases. Genetic analysis is an effective method to improve diagnostic certainty.

Additionally, in our study, the proportion of *APOE4* positive cases between FAD and SAD, EOAD and LOAD, were not significantly different, which is inconsistent with previous studies reporting that *APOE4* exerts its maximal effect in EOAD^55,56^. Perhaps other genetic or environmental factors may play an important role in the onset and pathogenesis of AD.

This study represents a comprehensive and systematic screening of 36 dementia-associated genes in AD, FTD, and DLB patients from South China, although the current study has some limitations. First, we only focused on known 36 dementia-associated genes, not susceptibility genes, risk loci, or new candidate genes, which may play important roles in neurodegenerative dementia. Second, in this study, we only screened neurodegenerative dementia patients, but no controls were assessed to compare background frequencies of the P/LP variants. Lastly, for those novel variants identified in this study, we did not design functional experiments to further validation.

In conclusion, we have conducted the most systematic survey of the mutational spectrum of neurodegenerative dementia patients in South Chinese population, which further expanded the mutational spectrum of dementia-related genes and have provided evidence that there is some genetic heterogeneity and perhaps overlap between phenotypes. Our results may prove to be beneficial for clinical prediction, diagnosis, and genetic counseling and may generate hypotheses for future basic research on genetic-associated pathogenesis of neurodegenerative dementia.

## Methods

### Study participants

A total of 1795 patients with neurodegenerative dementias, including 1592 with AD, 110 with FTD, and 93 with DLB, were recruited at the Xiangya Hospital, Central South University, between February 2004 and October 2020. All patients were unrelated probands. The demographic and clinical characteristics are summarized in Table 1. All subjects had been clinically diagnosed with AD, FTD, or DLB according to international guidelines. This study was approved by the Ethics Committee of Xiangya Hospital, Central South University, China. Written informed consent was obtained from each participant or guardian.

### Targeted genes sequencing

Genomic DNA was extracted from peripheral blood leukocytes of each participant using the QIAGEN kit according to the manufacturer’s instructions. We designed a dementia-related gene panel containing a total of 36 genes associated with cognitive impairment phenotypes, including *PSEN1*, *PSEN2*, *APP*, *APOE, ABCA7, SORL1, TREM2, ADAM10, MAPT, GRN, FUS, TARDBP, VCP, TBK1, CHCHD10, HTRA1, SQSTM1, UBQLN1, CHMP2B, SIGMAR1, OPTN, HNRNPA1, HNRNPA2B1, PRKAR1B, TMEM106B, UBQLN2*, *NOTCH3, TREX1, GLA, COL4A1, CSF1R, GBA, SNCA, SNCB, LRRK2*, and *PRNP*. Briefly, gDNA was fragmented and a paired-end library was constructed using Covaris LE220 (Massachusetts, USA), followed by pre-capture PCR amplification. After PCR amplification, the DNA fragments were captured by the targeted panel, followed by sequencing using the Illumina NovaSeq 6000 platform. The reads were mapped to the human genome reference (hg19) using the Burrows Wheeler Aligner software (http://bio-bwa.sourceforge.net)^57^, and duplicate sequence reads were removed using Picard (http://broadinstitute.github.io/picard/). Variant calling was performed using the Genome Analysis Toolkit (https://software.broadinstitute.org/gatk/)^58^. The variants were annotated using ANNOVAR (https://hpc.nih.gov/apps/ANNOVAR.html)^59^ and named according to the guidelines of the Human Genome Variation Society (http://www.hgvs.org/)^60^. Pathogenic or likely pathogenic (P/LP) variants were assessed according to the guidelines issued by the American College of Medical Genetics (ACMG).

Moreover, the (GGGGCC)n repeats in *C9orf72* and (CAG)n repeats in *HTT* were performed in all individuals using previously reported repeat-primed polymerase chain reaction and capillary electrophoresis^61,62^.

### Sanger sequencing

**Fig. 5:**
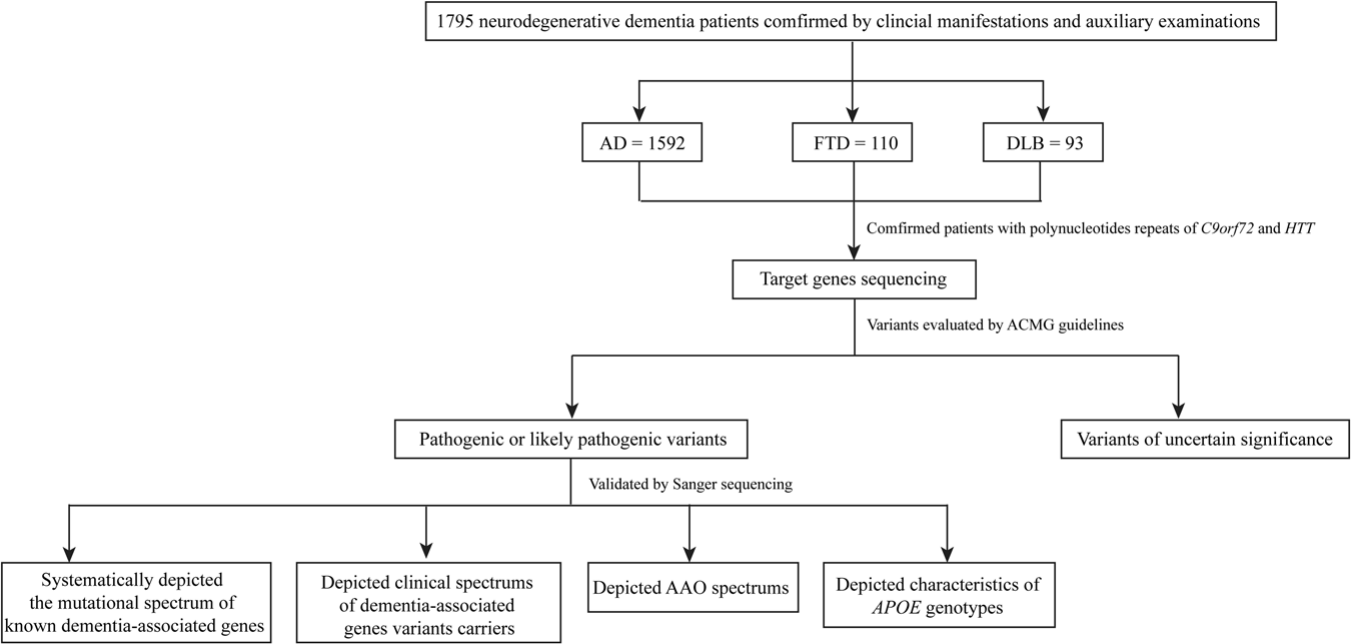
Workflow of this study. *AD* Alzheimer’s disease, *FTD* frontotemporal dementia, *DLB* dementia with Lewy bodies.

All P/LP variants were estimated by PCR amplification and Sanger sequencing using a Big Dye Terminator V3.1 on an ABI 3730xl DNA analyzer (Applied Biosystems, Foster City, USA). The DNA sequences were then analyzed using Sequencher software version 4.2. All primers were designed using Primer 5, and the primer sequences and PCR reaction conditions are listed in Supplementary Table 3. Meanwhile, variants of unknown significance identified in this study are shown in Supplementary Table 4. The study workflow is shown in Fig. 5.

### Statistical analysis

Quantitative variables such as age at onset, age at diagnosis, disease duration, education attainment, and cognitive assessment score are expressed as the mean ± SD. All data were tested for normality and homogeneity of variance using the Shapiro-Wilk test and Levene variance equality test. Two independent samples were conducted using the *t* test or the Mann–Whitney *U* test. The *χ*^2^ test and Fisher exact test were used to analyze categorical data, such as the proportion of female patients, family history, the percentage of *APOE4* positive or negative, and proportion of EOAD, LOAD or P/LP variants carriers and non-carriers patients. Multiple linear regression analysis was performed to correct the confounding factors and explore the factors affecting the AAO. All tests were two-tailed, and *p* < 0.05 was considered statistically significant. All analyses were performed using SPSS v.26 (IBM). Data were visualized using Prism 8 (GraphPad).

### Reporting summary

Further information on research design is available in the Nature Research Reporting Summary linked to this article.

## Supplementary information

Supplementary Information

Reporting Summary

## Data Availability

The sequencing raw data analyzed during this study has been deposited in European Variation Archive, the accession number was PRJEB46658. All other data are available from the corresponding authors on reasonable request.

## Code availability

No custom code or scripts were used for the generation or processing of datasets.
