# Supplementary material for: The role of genetics in neurodegenerative dementia: a large cohort study in South China
Source: NPJ Genom Med. 2021 Aug 13;6:69. doi: 10.1038/s41525-021-00235-3 (PMC8363644; doi:10.1038/s41525-021-00235-3)
Supplement: Supplementary file 1 — Supplementary Information [file 41525_2021_235_MOESM1_ESM.docx]

| **Supplementary Table 1**  36 genes associated with cognitive impairment phenotypes in the gene panel | | |
| --- | --- | --- |
| **Gene symbol** | **Full name** | **Related disease** |
| *PSEN1* | Presenilin 1 | Alzheimer’s disease (AD)^1^ |
| *PSEN2* | Presenilin 2 | AD^2^ |
| *APP* | Amyloid beta precursor protein | AD^3^ |
| *APOE* | Apolipoprotein E | AD^4^ |
| *ABCA7* | ATP binding cassette subfamily A member 7 | AD^5^ |
| *SORL1* | Sortilin related receptor 1 | AD^6^ |
| *TREM2* | Triggering receptor expressed on myeloid cells 2 | AD^7^; FTD^8^; Nasu-Hakola disease^9^ |
| *ADAM10* | ADAM metallopeptidase domain 10 | AD^10^; Reticulate acropigmentation of Kitamura^11^ |
| *MAPT* | Microtubule associated protein tau | Frontotemporal dementia (FTD)^12^; AD^13^; Corticobasal Degeneration (CBD); Dementia with Lewy Bodies (DLB); Parkinson's Disease Dementia (PDD); Pick's disease; Progressive Supranuclear Palsy (PSP)^14^ |
| *GRN* | Granulin precursor | FTD^15^; AD^16^ |
| *FUS* | FUS RNA binding protein | Amyotrophic lateral sclerosis (ALS)^17^ |
| *TARDBP* | TAR DNA binding protein | ALS; FTD^18^ |
| *VCP* | Valosin containing protein | Inclusion body myopathy associated with Paget disease of bone and frontotemporal dementia (IBMPFD); FTD^19^ |
| *TBK1* | TANK binding kinase 1 | FTD; ALS^20^ |
| *CHCHD10* | Coiled-coil-helix-coiled-coil-helix domain containing 10 | FTD; ALS^21^ |
| *HTRA1* | HtrA serine peptidase 1 | Cerebral autosomal recessive arteriopathy with subcortical infarcts and leukoencephalopathy (CARASIL)^22^ |
| *SQSTM1* | Sequestosome 1 | Paget disease of bone^23^; FTD^24^; ALS^25^ |
| *UBQLN1* | Ubiquilin 1 | AD^26^ |
| *CHMP2B* | Charged multivesicular body protein 2B | FTD^27^; ALS^28^ |
| *SIGMAR1* | Sigma non-opioid intracellular receptor 1 | ALS^29^; FTD- Motor neuron disease (MND)^30^ |
| *OPTN* | Optineurin | FTD^31^; ALS^32^ |
| *HNRNPA1* | Heterogeneous nuclear ribonucleoprotein A1 | ALS^33^; IBMPFD^34^ |
| *HNRNPA2B1* | Heterogeneous nuclear ribonucleoprotein A2/B1 | IBMPFD^34^ |
| *PRKAR1B* | Protein kinase cAMP-dependent type I regulatory subunit beta | Neuronal intermediate filament inclusion disease^35^ |
| *TMEM106B* | Transmembrane protein 106B | FTD^36^ |
| *UBQLN2* | Ubiquilin 2 | ALS; ALS-FTD^37^ |
| *NOTCH3* | Notch receptor 3 | Cerebral autosomal dominant arteriopathy with subcortical infarcts and leukoencephalopathy (CADASIL)^38^ |
| *TREX1* | Three prime repair exonuclease 1 | Vasculopathy, retinal, with cerebral leukodystrophy (RVCL)^39^ |
| *GLA* | Galactosidase alpha | Fabry disease (FD)^40^ |
| *COL4A1* | Collagen type IV alpha 1 chain | Cerebral small vessel disease^41^; Intracerebral hemorrhage (ICH)^42^ |
| *CSF1R* | Colony stimulating factor 1 receptor | Hereditary diffuse leukoencephalopathy with spheroids (HDLS)^43^ |
| *GBA* | Glucosylceramidase beta | Parkinson's disease (PD)^44^ |
| *SNCA* | Synuclein alpha | Dementia with Lewy body (DLB)^45^ |
| *SNCB* | Synuclein beta | DLB^46^ |
| *LRRK2* | Leucine rich repeat kinase 2 | PD^47^ |
| *PRNP* | Prion protein | Creutzfeldt-Jakob disease^48^ |

| **Supplementary Table 2**  Pathogenic or likely pathogenic variants identified in this study | | | | | | | | | | | | |
| --- | --- | --- | --- | --- | --- | --- | --- | --- | --- | --- | --- | --- |
| **No. of**  **cases** | **Gene** | **Mutation (CDS)** | **Protein level** | **Software prediction** | | | **Allele frequency in population** | | | | | |
|  |  |  |  | **SIFT** | **Polyphen2** | **Mutation Taster** | **gnomAD ALL** | **gnomAD EAS** | **ExAC ALL** | **1000 genomes ALL** | **mBioBank** | **ACMG** |
| 1 | *PSEN1* | c.250A>G | p.M84V | 0.083/T | 0.996/D | Disease_causing | - | - | - | - | - | P (PS1+PS3+PM1+PM2+PP1+PP2+PP5) |
| 1 | *PSEN1* | c.415A>G | p.M139V | 0.174/T | 0.992/D | Disease_causing | - | - | - | - | - | P (PS1+PS3+PM1+PM2+PM5+PP2+PP5) |
| 1 | *PSEN1* | c.415A>T | p.M139L | 0.685/T | 0.982/D | Disease_causing | - | - | - | - | - | P (PS1+PS3+PM1+PM2+PM5+PP2+PP5) |
| 2 | *PSEN1* | c.424G>A | p.V142I | 0.089/T | 0.999/D | Disease_causing | - | - | - | - | - | P (PS1+PM1+PM2+PM5+PP2) |
| 1 | *PSEN1* | c.436A>G | p.M146V | 0.012/D | 0.992/D | Disease_causing | - | - | - | - | - | P (PS1+PS3+PM1+PM2+PM5+PP1+PP2+PP3) |
| 1 | *PSEN1* | c.451G>A | p.V151M | 0.001/D | 1/D | Disease_causing | - | - | - | - | - | LP (PM1+PM2+PP2+PP3) |
| 1 | *PSEN1* | c.519G>T | p.L173F | 0.001/D | 0.996/D | Disease_causing | - | - | - | - | - | P (PS1+PS3+PM1+PM2+PM5+PP1+PP2+PP3) |
| 1 | *PSEN1* | c.604A>T | p.I202F | 0.008/D | 0.978/D | Disease_causing | 4.06e-06 | 0 | 8.237e-06 | - | - | P (PS3+PM1+PP1+PP2+PP3+PP5) |
| 1 | *PSEN1* | c.617G>A | p.G206D | 0.001/D | 1/D | Disease_causing | - | - | - | - | - | P (PS1+PS3+PM1+PM2+PM5+PP1+PP2+PP3+PP5) |
| 1 | *PSEN1* | c.677T>G | p.L226R | 0/D | 1/D | Disease_causing | - | - | - | - | - | P (PS1+PM1+PM2+PM5+PP2+PP3) |
| 1 | *PSEN1* | c.679A>C | p.I227L | 0.003/D | 0.998/D | Disease_causing | - | - | - | - | - | P (PS1+PM1+PM2+PM5+PP2+PP3+PP5) |
| 1 | *PSEN1* | c.697A>G | p.M233V | 0.098/T | 0.995/D | Disease_causing | - | - | - | - | - | P (PS3+PM1+PM2+PM5+PP1+PP2+PP3+PP5) |
| 2 | *PSEN1* | c.791C>T | p. P264L | 0.002/D | 1/D | Disease_causing | 4.08e-06 | 0 | - | - | - | P (PS1+PS3+PM1+PM2+PP1+PP2+PP3+PP5) |
| 2 | *PSEN1* | c.806G>A | p.R269H | 0.001/D | 1/D | Disease_causing | 4.07e-06 | 0 | 8.701e-06 | - | - | P (PS1+PM1+PM5+PP2+PP3+PP5) |
| 1 | *PSEN1* | c.845T>G | p.L282R | 0.001/D | 0.998/D | Disease_causing | - | - | - | - | - | P (PS3+PM1+PM2+PM5+PP2+PP3+PP5) |
| 1 | *PSEN1* | c.854C>T | p.A285V | 0.015/D | 1/P | Disease_causing | - | - | - | - | - | LP (PM1+PM2+PM5+PP2+PP3+PP5) |
| 1 | *PSEN1* | c.1139A>G | p.K380R | 0.078/T | 0.999/D | Disease_causing | - | - | - | - | - | LP (PM1+PM2+PP2+PP3) |
| 1 | *PSEN1* | c.1174C>G | p.L392V | 0.002/D | 0.995/D | Disease_causing | - | - | - | - | - | LP (PS3+PM1+PM2+PP2+PP3) |
| 1 | *PSEN1* | c.1369A>G | p.M457V | 0.337/T | 0.987/D | Disease_causing | - | - | - | - | - | P (PS1+PM1+PM2+PP2+PP3) |
| 4 | *PSEN2* | c.715A>G | p.M239V | 0.089/T | 0.846/D | Disease_causing | - | - | - | - | - | LP (PM2+PM5+PP2+PP3+PP5) |
| 1 | *PSEN2* | c.716T>C | p.M239T | 0.026/D | 1/D | Disease_causing | - | - | - | - | - | LP (PM2+PM5+PP2+PP3) |
| 1 | *PSEN2* | c.717G>A | p.M239I | 0.08/T | 0.993/D | Disease_causing | - | - | - | - | - | LP  (PM2+PM5+PP2+PP5) |
| 1 | *PSEN2* | c.1180delG | p.A394Pfs*8 |  |  |  | - | - | - | - | - | LP (PVS1+PM2) |
| 1 | *APP* | c.2143G>A | p.V715M | 0.001/D | 0.999/D | Disease_causing | - | - | - | - | - | P (PS3+PM1+PM2+PM5+PP1+PP3+PP5) |
| 3 | *APP* | c.2149G>A | p.V717I | 0.167/T | 1/D | Disease_causing | - | - | - | - | - | P (PS3+PM1+PM2+PM5+PP1+PP3+PP5) |
| 1 | *MAPT* | c.1788T>G | p.N596K | 0.013/D | 0.999/D | Disease_causing | - | - | - | - | - | LP (PM2+PM5+PP2+PP3) |
| 1 | *MAPT* | c.1907C>T | p.P636L | 0.0/D | 1/D | Disease_causing | 5.544e-06 | 0 | - | - | - | LP (PM2+PM5+PP2+PP3) |
| 1 | *GRN* | c.20G>A | p.W7* | - | - | Disease_causing | - | - | - | - | - | P (PVS1+PM1+PM2+PP3) |
| 1 | *GRN* | c.328C>T | p.R110* | - | - | Disease_causing | 4.074e-06 | 0 | 8.988e-06 | - | - | P (PVS1+PM1+PP3) |
| 1 | *CHCHD10* | c.121C>T | p.Q41* | - | - | Disease_causing | - | - | - | - | - | P (PVS1+PM1+PM2+PP3) |
| 1 | *CHCHD10* | c.283C>T | p.Q95* | - | - | Disease_causing | - | - | - | - | - | P (PVS1+PM1+PM2+PP3) |
| 1 | *HTRA1* | c.589C>T | p.R197* | - | - | Disease_causing | 8.121e-06 | 0 | - | - | - | P (PVS1+PM1+PM2+PP3) |
| 1 | *OPTN* | c.1402_1407del | p.468_469del | - | - | - | - | - | - | - | - | LP (PM1+PM4+PM2+PP3) |
| 1 | *SQSTM1* | c.558_559insC | p.V287Rfs*21 |  |  |  | - |  | - |  | - | LP (PVS1+PM2) |
| 1 | *VCP* | c.475C>T | p.R159C | 0.001/D | 1/D | Disease_causing | 4.06e-06 | 0 | 8.25e-06 | - | - | LP (PS1+PM5+PP2+PP3) |
| 1 | *SIGMAR1* | c.26G>A | p.W9* | 0.003/D | 0.98/D | Disease_causing | - | - | - | - | - | P (PVS1+PM2+PP2+PP3) |
| 1 | *TBK1* | c.973dup | p.Y325Lfs*4 | - | - | - | - | - | - | - | - | P  (PVS1+PM1+PM2) |
| 1 | *C9orf72* | Hexanucleotide expansion | **-** | **-** | **-** | **-** | **-** | **-** | **-** | **-** | **-** |  |
| 1 | *HTT* | CAG repeat expansions | - | **-** | **-** | **-** | **-** | **-** | **-** | **-** | **-** |  |

D: damaging; T: tolerable; B = benign; P: pathogenic; LP: likely pathogenic.

| **Supplementary Table 3**  PCR conditions and primer sequences of *PSEN1/2*, *APP*, *MAPT*, *GRN*, *CHCHD10*, *TBK1*, *HTRA1*, *OPTN*, *SQSTM1*, *VCP*, *SIGMAR1* and *APOE* | | |
| --- | --- | --- |
| ***PSEN1* exon** | **Primer sequence** | **Size in bp** |
| Exon4 | F: CGTTACCTTGATTCTGCTGA  R: GACATGCTGTAAAGAAAAGCC | 371 |
| Exon5 | F: TCTTAGCTAGATTGGTGAGTTGG  R: AATGTTCCACAGTGAGGAGGAAG | 443 |
| Exon6 | F: GGCATAGTGGTGCACATCTGTAAT  R: GAGCTTCACTATTCTCTTTGACAG | 879 |
| Exon7 | F: GGAGCCATCACATTATTCTAAA  R: AACAAATTATCAGTCTTGGGTTT | 326 |
| Exon8 | F: CTTCGTTAATTCCTCCCTAC  R: AGTTCCAGGAATGCTGTG | 307 |
| Exon9 | F: TGTGTGTCCAGTGCTTACCTG  R: TGTTAGCTTATAACAGTGACCCTG | 188 |
| Exon10 | F: CCAGCTAGTTACAATGACAGC  R: TCAAAAAGGTTGATAATGTAGCT | 345 |
| Exon11 | F: GGTTGAGTAGGGCAGTGATA  R: TTAAAGGGACTGTGTAATCAAAG | 275 |
| Exon12 | F: GTCTTTCCCATCTTCTCCAC  R: GGGATTCTAACCGCAAATAT | 199 |
| ***PSEN2* exon** |  |  |
| Exon3 | F: GTCCTCCACTGCCTTTGTCTCAC  R: CTTCCCTTCTCCCTCCCGCATCAG | 329 |
| Exon4 | F: GGAAAGCAACATTCAAACTTCTC  R: CAAGTAGGTCACAATCCAGGAG | 506 |
| Exon5 | F: GGAGGACAGGAACTGCTCAT  R: TGGGTCTATTTTCCTCTAATTTGTG | 427 |
| Exon6 | F: CCAGCGTAGGCATGAAGT  R: TTCCCACAAACCAGAAAGTA | 536 |
| Exon7 | F: GAAGGTCGGGGAAGGAAAT  R: CTCTGTTTTACAAAGGCGA | 499 |
| Exon8 | F: TGTGACTGGAGAATGAGAATTTGG  R: CCCTGGCTTCTGAAAGTGGAG | 383 |
| Exon9 | F: CAACGGCCTCCTAACAATG  R: CAACGGACCTTTCCCTCT | 364 |
| Exon10 | F: CTCTGACCAGCTGTTGTTTC  R: AGCCTCCACCCTCTGTCT | 249 |
| Exon11 | F: TTCCATTCTGTGCACGCCTC  R: ACCTGCCCCCACCACAATG | 244 |
| Exon12 | F: ACACCAGGGATCACCACGCTCAC  R: TGCCTCCTCCTCACCAAGTAAACA | 344 |
| ***APP* exon** |  |  |
| Exon16 | F: TTCAGGCCTAGAAAGAAGTTTTG  R: CAGCCTAGCCTATTTATTTTCTTCA | 370 |
| Exon17 | F: CCTCATCCAAATGTCCCCTGCATT  R: CCACTTGGAAACATGCAGTCAAGTT | 293 |
| ***MAPT****: c.1788T>G, p.N596K* | F: CTCTGCCAAGTCCGAAAGTG  R: ATCCTGAGAGCCCAAGAAGG | 361 |
| ***MAPT****: c.1907C>T, p.P636L* | F: GAAAGTGGAGGCGTCCTTG  R: AATATGAGGAAGGGGCTTCTG | 374 |
| ***GRN****: c.328C>T, p.R110** | F: ACTCTCAGCCCCTGCAGAT  R: CAAGATGACCCTTTGGGAAA | 371 |
| ***CHCHD10****: c. 121C>T, p.Q41** | F: ACGTGGGTCGTCCATCTCT  R: CCTGCCTCAGTTTCTCTTGG | 352 |
| ***CHCHD10****: c.283C>T, p.Q95** | F: AAAAGGCAGCAGAAACATGC  R: AAGAGGAGGGTTGGCCTCT | 383 |
| ***TBK1****: c.973dup,* *p.Y325Lfs*4* | F: CAGTTCCTTTGATTTGCTGGT  R: TGGGAAATGGGTACTTTTGG | 389 |
| ***HTRA1****: c.589C>T, p.R197** | F: AGCGATGGCTAGGTGTGTGT  R: AGAGAGCTCTGGTGCCTTTG | 387 |
| ***OPTN****: c.1402_1407del, p.468_469del* | F: AGCAGGATTGTGCATCTGTG  R: ACCTAGGCAGGAGTGCAGTG | 389 |
| ***SQSTM1****: c.558_559insC, p.V287Rfs*21* | F: GTAGCGTCTGCGAGGGAAAG  R: AGGCTGCCTGACTACTGTCAC | 369 |
| ***VCP****: c.475C>T, p.R195C* | F: GGTGGAGTTGGGGAGAGGTA  R: CCCAAAGTACTGGGATTACAGG | 400 |
| ***SIGMAR1****: c.26G>A, p.W9** | F: AGGAAATGGTTCAACCGAAG  R: GAGAGAAGGCCAGCTCGTG | 489 |
| ***APOE* genotype** | F: CCTACAAATCGGAACTGG  R: CTCGAACCAGCTCTTGAG | 581 |

| **Supplementary Table 4**  Variants of unknown significance identified in this study | | |
| --- | --- | --- |
| **Gene symbol** | **Base change** | **Protein change** |
| ***PSEN1*** | c.932A>G | p.K311R |
|  | c.679A>C | p.I227L |
|  | c.808A>T | p.M270L |
| ***PSEN2*** | c.49C>T | p.R17W |
|  | c.70G>A | p.E24K |
|  | c.100G>A | p.G34S |
|  | c.208G>A | p.G70R |
|  | c.382A>G | p.T128A |
|  | c.409A>T | p.N137Y |
|  | c.437T>C | p.I146T |
|  | c.505C>A | p.H169N |
|  | c.640G>T | p.V214L |
|  | c.793G>A | p.V265M |
|  | c.893T>A | p.M298K |
|  | c.1056_1064del | p.352_355del |
|  | IVS11: c.1073-2_1073-1del | - |
|  | c.1094G>C | p.G365A |
|  | c.1304G>A | p.R435Q |
| ***APP*** | c.62C>T | p.P21L |
|  | c.77C>G | p.A26G |
|  | c.475A>G | p.S159G |
|  | c.890C>T | p.T297M |
|  | c.896C>G | p.P299R |
|  | c.995A>G | p.D332G |
|  | c.1024G>A | p.G342S |
|  | c.1450C>T | p.P484S |
|  | c.1463G>A | p.R488H |
|  | c.1549A>C | p.M517L |
|  | c.1579C>T | p.R527W |
|  | c.1943G>A | p.R648Q |
| ***MAPT*** | c.14G>T | p.R5H |
|  | c.272C>T | p.A91V |
|  | c.418C>T | p.P140S |
|  | c.530A>T | p.D177V |
|  | c.797C>T | p.P266L |
|  | c.1343G>A | p.R448Q |
|  | c.1382G>A | p.G461E |
|  | c.1537C>G | p.P513A |
|  | c.1583A>T | p.D528V |
|  | c.1736A>C | p.Q579P |
|  | c.1842T>G | p.N614K |
| ***GRN*** | c.299C>T | p.P100L |
|  | c.943T>C | p.C315R |
|  | c.1327G>A | p.G443S |
|  | c.1373C>A | p.P458Q |
|  | c.1460C>T | p.T487I |
|  | c.1555G>A | p.V519M |
|  | c.1690C>T | p.R564C |
| ***CHCHD10*** | c.66C>A | p.H22Q |
|  | c.275A>G | p.Y92C |
|  | c.406dupC | p.H136Pfs*63 |
| ***FUS*** | c.52C>A | p.P18T |
|  | c.448C>T | p.P150S |
|  | c.667_668insGCT | p.G223delinsGC |
|  | c.1173G>A | p.M391I |
|  | c.1534G>A | p.D512N |
| ***HNPNPA1*** | c.191C>G | p.T64S |
|  | c.691A>G | p.S231G |
|  | c.847G>A | p.G283R |
| ***GBA*** | c.1156T>C | p.F386L |
| ***ABCA7*** | c.1374delC | p.G460Afs*34 |
|  | c.3605delG | p.G1202Afs*15 |
|  | c.1832T>G | p.V611G |
|  | c.898G>A | p.G300S |
| ***OPTN*** | c.76C>A | p.H26N |
|  | c.77A>G | p.H26R |
|  | c.263T>C | p.I88T |
|  | c.407C>T | p.A136V |
|  | c.523G>A | p.E175K |
|  | c.909C>A | p.N303K |
|  | c.1184A>G | p.K395R |
|  | c.1319A>G | p.K440R |
|  | c.1481T>G | p.L494W |
|  | c.1633C>T | p.R545W |
| ***PRKAR1B*** | c.200C>T | p.A67V |
|  | c.215A>G | p.N72S |
|  | c.899C>T | p.A300V |
|  | c.914G>A | p.R305H |
|  | c.937G>A | p.V313M |
| ***SIGMAR1*** | c.295A>C | p.I99L |
|  | c.310G>A | p.V104M |
|  | c.320T>C | p.F107S |
|  | c.430G>A | p.V144I |
|  | c.558_559insC | p.V287Rfs*21 |
|  | c.565A>G | p.T189A |
|  | c.887C>T | p.P296L |
|  | c.907G>A | p.A303T |
|  | c.911C>T | p.T304M |
|  | c.923C>T | p.A308V |
|  | c.943G>A | p.A315T |
|  | c.1316C>T | p.P439L |
| ***TBK1*** | c.34T>G | p.S12A |
|  | c.1261G>C | p.V421L |
|  | c.1631C>T | p.P544L |
| ***TMEM106B*** | c.434A>T | p.N145I |
| ***UBQLN1*** | c.104A>G | p.K35R |
|  | c.1130T>C | p.M377T |
| ***VCP*** | c.555A>C | p.E185D |
|  | c.814C>T | p.P272S |
| ***SORL1*** | c.6586G>A | p.D2196N |
|  | c.1953C>A | p.F651L |

**References**

1. Group, A.D.C. The structure of the presenilin 1 (S182) gene and identification of six novel mutations in early onset AD families. *Nat Genet* **11**, 219-222 (1995).

2. Rogaev, E.I., Sherrington, R., Rogaeva, E.A., Levesque, G. & Ikeda, M. et al. Familial Alzheimer's disease in kindreds with missense mutations in a gene on chromosome 1 related to the Alzheimer's disease type 3 gene. *Nature* **376**, 775-778 (1995).

3. A Goate, M.C.C.M. Segregation of a missense mutation in the amyloid precursor protein gene with familial Alzheimer's disease. *Nature* **6311**, 704-706 (1991).

4. J Poirier, J.D.D.B. Apolipoprotein E polymorphism and Alzheimer's disease. *Lancet* **342**, 697-699 (1993).

5. Hollingworth, P., Harold, D., Sims, R., Gerrish, A. & Lambert, J. et al. Common variants at ABCA7, MS4A6A/MS4A4E, EPHA1, CD33 and CD2AP are associated with Alzheimer's disease. *Nat Genet* **43**, 429-435 (2011).

6. Rogaeva, E., Meng, Y., Lee, J.H., Gu, Y. & Kawarai, T. et al. The neuronal sortilin-related receptor SORL1 is genetically associated with Alzheimer disease. *Nat Genet* **39**, 168-177 (2007).

7. Jonsson, T., Stefansson, H., Steinberg, S., Jonsdottir, I. & Jonsson, P.V. et al. Variant of TREM2 associated with the risk of Alzheimer's disease. *New Engl J Med* **368**, 107-116 (2013).

8. Guerreiro, R.J., Lohmann, E., Bras, J.M., Gibbs, J.R. & Rohrer, J.D. et al. Using exome sequencing to reveal mutations in TREM2 presenting as a frontotemporal dementia-like syndrome without bone involvement. *JAMA Neurol* **70**, 78-84 (2013).

9. Pekkarinen, P., Salminen, A., Phillips, J.H., Peltonen, L. & Böhling, T. et al. Loss-of-function mutations in TYROBP ( DAP12 ) result in a presenile dementia with bone cysts. *Nat Genet* **25**, 357-361 (2000).

10. Kim, M., Suh, J., Romano, D., Truong, M.H. & Mullin, K. et al. Potential late-onset Alzheimer's disease-associated mutations in the ADAM10 gene attenuate α-secretase activity. *Hum Mol Genet* **18**, 3987-3996 (2009).

11. Kono, M., Sugiura, K., Suganuma, M., Hayashi, M. & Takama, H. et al. Whole-exome sequencing identifies ADAM10 mutations as a cause of reticulate acropigmentation of Kitamura, a clinical entity distinct from Dowling-Degos disease. *Hum Mol Genet* **22**, 3524-3533 (2013).

12. Poorkaj, P., Bird, T.D., Wijsman, E., Nemens, E. & Garruto, R.M. et al. Tau is a candidate gene for chromosome 17 frontotemporal dementia. *Ann Neurol* **43**, 815-25 (1998).

13. Jin, S.C., Pastor, P., Cooper, B., Cervantes, S. & Benitez, B.A. et al. Pooled-DNA sequencing identifies novel causative variants in PSEN1, GRN and MAPT in a clinical early-onset and familial Alzheimer's disease Ibero-American cohort. *Alzheimers Res Ther* **4**, 34-34 (2012).

14. Poorkaj, P., Muma, N.A., Zhukareva, V., Cochran, E.J. & Shannon, K.M. et al. An R5L tau mutation in a subject with a progressive supranuclear palsy phenotype. *Ann Neurol* **52**, 511-516 (2002).

15. Baker, M., Mackenzie, I.R., Pickering-Brown, S.M., Gass, J. & Rademakers, R. et al. Mutations in progranulin cause tau-negative frontotemporal dementia linked to chromosome 17. *Nature* **442**, 916-919 (2006).

16. Brouwers, N., Sleegers, K., Engelborghs, S., Maurer-Stroh, S. & Gijselinck, I. et al. Genetic variability in progranulin contributes to risk for clinically diagnosed Alzheimer disease. *Neurology* **71**, 656-664 (2008).

17. Kwiatkowski, T.J., Bosco, D.A., Leclerc, A.L., Tamrazian, E. & Vanderburg, C.R. et al. Mutations in the FUS/TLS gene on chromosome 16 cause familial amyotrophic lateral sclerosis. *Science* **323**, 1205-1208 (2009).

18. Van Deerlin, V.M., Leverenz, J.B., Bekris, L.M., Bird, T.D. & Yuan, W. et al. TARDBP mutations in amyotrophic lateral sclerosis with TDP-43 neuropathology: a genetic and histopathological analysis. *Lancet Neurol* **7**, 409-416 (2008).

19. Guyant-Marechal, L., Laquerriere, A., Duyckaerts, C., Dumanchin, C. & Bou, J. et al. Valosin-containing protein gene mutations: clinical and neuropathologic features. *Neurology* **67**, 644-51 (2006).

20. Freischmidt, A., Wieland, T., Richter, B., Ruf, W. & Schaeffer, V. et al. Haploinsufficiency of TBK1 causes familial ALS and fronto-temporal dementia. *Nat Neurosci* **18**, 631-636 (2015).

21. Bannwarth, S., Ait-El-Mkadem, S., Chaussenot, A., Genin, E.C. & Lacas-Gervais, S. et al. A mitochondrial origin for frontotemporal dementia and amyotrophic lateral sclerosis through CHCHD10 involvement. *Brain* **137**, 2329-2345 (2014).

22. Hara, K., Shiga, A., Fukutake, T., Nozaki, H. & Miyashita, A. et al. Association of HTRA1 mutations and familial ischemic cerebral small-vessel disease. *N Engl J Med* **360**, 1729-1739 (2009).

23. Laurin, N., Brown, J.P., Morissette, J. & Raymond, V. Recurrent mutation of the gene encoding sequestosome 1 (SQSTM1/p62) in Paget disease of bone. *Am J Hum Genet* **70**, 1582-1588 (2002).

24. Le Ber, I. SQSTM1 mutations in French patients with frontotemporal dementia or frontotemporal dementia with amyotrophic lateral sclerosis. *JAMA Neurol* **70**, 1403-1410 (2013).

25. Fecto, F., Yan, J., Vemula, S.P., Liu, E. & Yang, Y. et al. SQSTM1 mutations in familial and sporadic amyotrophic lateral sclerosis. *Arch Neurol* **68**, 1440-1446 (2011).

26. Bertram, L., Hiltunen, M., Parkinson, M., Ingelsson, M. & Lange, C. et al. Family-Based Association between Alzheimer's Disease and Variants in UBQLN1. *N Engl J Med* **352**, 884-894 (2005).

27. Skibinski, G., Parkinson, N.J., Brown, J.M., Chakrabarti, L. & Lloyd, S.L. et al. Mutations in the endosomal ESCRTIII-complex subunit CHMP2B in frontotemporal dementia. *Nat Genet* **37**, 806-808 (2005).

28. Parkinson, N., Ince, P.G., Smith, M.O., Highley, R. & Skibinski, G. et al. ALS phenotypes with mutations in CHMP2B (charged multivesicular body protein 2B). *Neurology* **67**, 1074-1077 (2006).

29. Al-Saif, A., Al-Mohanna, F. & Bohlega, S. A mutation in sigma-1 receptor causes juvenile amyotrophic lateral sclerosis. *Ann Neurol* **70**, 913-919 (2011).

30. Luty, A.A., Kwok, J.B., Dobson-Stone, C., Loy, C.T. & Coupland, K.G. et al. Sigma nonopioid intracellular receptor 1 mutations cause frontotemporal lobar degeneration-motor neuron disease. *Ann Neurol* **68**, 639-649 (2010).

31. Pottier, C., Bieniek, K.F., Finch, N., van de Vorst, M. & Baker, M. et al. Whole-genome sequencing reveals important role for TBK1 and OPTN mutations in frontotemporal lobar degeneration without motor neuron disease. *Acta Neuropathol* **130**, 77-92 (2015).

32. Maruyama, H., Morino, H., Ito, H., Izumi, Y. & Kato, H. et al. Mutations of optineurin in amyotrophic lateral sclerosis. *Nature* **465**, 223-226 (2010).

33. Liu, Q., Shu, S., Wang, R.R., Liu, F. & Cui, B. et al. Whole-exome sequencing identifies a missense mutation in hnRNPA1 in a family with flail arm ALS. *Neurology* **87**, 1763-1769 (2016).

34. Kim, H.J., Kim, N.C., Wang, Y., Scarborough, E.A. & Moore, J. et al. Mutations in prion-like domains in hnRNPA2B1 and hnRNPA1 cause multisystem proteinopathy and ALS. *Nature* **495**, 467-473 (2013).

35. Pottier, C., Baker, M., Dickson, D.W. & Rademakers, R. PRKAR1B mutations are a rare cause of FUS negative neuronal intermediate filament inclusion disease. *Brain* **138**, e357-e357 (2015).

36. Van Deerlin, V.M., Sleiman, P.M.A., Martinez-Lage, M., Chen-Plotkin, A. & Wang, L. et al. Common variants at 7p21 are associated with frontotemporal lobar degeneration with TDP-43 inclusions. *Nat Genet* **42**, 234-239 (2010).

37. Deng, H., Chen, W., Hong, S., Boycott, K.M. & Gorrie, G.H. et al. Mutations in UBQLN2 cause dominant X-linked juvenile and adult-onset ALS and ALS/dementia. *Nature* **477**, 211-215 (2011).

38. Joutel, A., Vahedi, K., Corpechot, C., Troesch, A. & Chabriat, H. et al. Strong clustering and stereotyped nature of Notch3 mutations in CADASIL patients. *Lancet* **350**, 1511-5 (1997).

39. Richards, A., van den Maagdenberg, A.M.J.M., Jen, J.C., Kavanagh, D. & Bertram, P. et al. C-terminal truncations in human 3′-5′ DNA exonuclease TREX1 cause autosomal dominant retinal vasculopathy with cerebral leukodystrophy. *Nat Genet* **39**, 1068-1070 (2007).

40. Sakuraba, H., Oshima, A., Fukuhara, Y., Shimmoto, M. & Nagao, Y. et al. Identification of point mutations in the alpha-galactosidase A gene in classical and atypical hemizygotes with Fabry disease. *Am J Hum Genet* **47**, 784-789 (1990).

41. Verdura, E., Hervé, D., Bergametti, F., Jacquet, C. & Morvan, T. et al. Disruption of a miR-29 binding site leading toCOL4A1 upregulation causes pontine autosomal dominant microangiopathy with leukoencephalopathy. *Ann Neurol* **80**, 741-753 (2016).

42. Weng, Y.C., Sonni, A., Labelle-Dumais, C., de Leau, M. & Kauffman, W.B. et al. COL4A1 mutations in patients with sporadic late-onset intracerebral hemorrhage. *Ann Neurol* **71**, 470-7 (2012).

43. Rademakers, R., Baker, M., Nicholson, A.M., Rutherford, N.J. & Finch, N. et al. Mutations in the colony stimulating factor 1 receptor (CSF1R) gene cause hereditary diffuse leukoencephalopathy with spheroids. *Nat Genet* **44**, 200-205 (2012).

44. Aharon-Peretz, J., Rosenbaum, H. & Gershoni-Baruch, R. Mutations in the Glucocerebrosidase Gene and Parkinson's Disease in Ashkenazi Jews. *N Engl J Med* **351**, 1972-1977 (2004).

45. Zarranz, J.J., Alegre, J., Gomez-Esteban, J.C., Lezcano, E. & Ros, R. et al. The new mutation, E46K, of alpha-synuclein causes Parkinson and Lewy body dementia. *Ann Neurol* **55**, 164-73 (2004).

46. Ohtake, H., Limprasert, P., Fan, Y., Onodera, O. & Kakita, A. et al. Beta-synuclein gene alterations in dementia with Lewy bodies. *Neurology* **63**, 805-811 (2004).

47. Zimprich, A., Biskup, S., Leitner, P., Lichtner, P. & Farrer, M. et al. Mutations in LRRK2 Cause Autosomal-Dominant Parkinsonism with Pleomorphic Pathology. *Neuron* **44**, 601-607 (2004).

48. Goldfarb, L.G., Petersen, R.B., Tabaton, M., Brown, P. & LeBlanc, A.C. et al. Fatal Familial Insomnia and Familial Creutzfeldt-Jakob Disease: Disease Phenotype Determined by a DNA Polymorphism. *Science* **258**, 806-808 (1992).
