# Supplementary material for: The role of genetics in neurodegenerative dementia: a large cohort study in South China
Source: NPJ Genom Med. 2021 Aug 13;6:69. doi: 10.1038/s41525-021-00235-3 (PMC8363644; doi:10.1038/s41525-021-00235-3)
Supplement: Supplementary file 2 — Reporting Summary [file 41525_2021_235_MOESM2_ESM.pdf]

nature research

Corresponding author(s): Lu Shen

Last updated by author(s): Jul 28, 2021

Reporting Summary

Nature Research wishes to improve the reproducibility of the work that we publish. This form provides structure for consistency and transparency in reporting. For further information on Nature Research policies, see our Editorial Policies and the Editorial Policy Checklist.

Statistics

For all statistical analyses, confirm that the following items are present in the figure legend, table legend, main text, or Methods section.

n/a	Confirmed	
☐	☒	The exact sample size (*n*) for each experimental group/condition, given as a discrete number and unit of measurement
☐	☒	A statement on whether measurements were taken from distinct samples or whether the same sample was measured repeatedly
☐	☒	The statistical test(s) used AND whether they are one- or two-sided<br>*Only common tests should be described solely by name; describe more complex techniques in the Methods section.*
☐	☒	A description of all covariates tested
☐	☒	A description of any assumptions or corrections, such as tests of normality and adjustment for multiple comparisons
☐	☒	A full description of the statistical parameters including central tendency (e.g. means) or other basic estimates (e.g. regression coefficient) AND variation (e.g. standard deviation) or associated estimates of uncertainty (e.g. confidence intervals)
☐	☒	For null hypothesis testing, the test statistic (e.g. *F*, *t*, *r*) with confidence intervals, effect sizes, degrees of freedom and *P* value noted<br>*Give P values as exact values whenever suitable.*
☒	☐	For Bayesian analysis, information on the choice of priors and Markov chain Monte Carlo settings
☐	☒	For hierarchical and complex designs, identification of the appropriate level for tests and full reporting of outcomes
☒	☐	Estimates of effect sizes (e.g. Cohen's *d*, Pearson's *r*), indicating how they were calculated

Our web collection on statistics for biologists contains articles on many of the points above.

Software and code

Policy information about availability of computer code

Data collection	ABI 3730xl DNA analyzer (Applied Biosystems, Foster City, USA), Illumina NovaSeq 6000 platform
Data analysis	SPSS v.26 (IBM), Prism 8 (GraphPad).

For manuscripts utilizing custom algorithms or software that are central to the research but not yet described in published literature, software must be made available to editors and reviewers. We strongly encourage code deposition in a community repository (e.g. GitHub). See the Nature Research guidelines for submitting code & software for further information.

Data

Policy information about availability of data

All manuscripts must include a data availability statement. This statement should provide the following information, where applicable:

- Accession codes, unique identifiers, or web links for publicly available datasets
- A list of figures that have associated raw data
- A description of any restrictions on data availability

The sequencing raw data analyzed during this study has been deposited in European Variation Archive, the accession number was PRJEB46658. All other data are available from the corresponding authors on reasonable request.

Field-specific reporting

Please select the one below that is the best fit for your research. If you are not sure, read the appropriate sections before making your selection.

☒ Life sciences ☐ Behavioural & social sciences ☐ Ecological, evolutionary & environmental sciences

For a reference copy of the document with all sections, see nature.com/documents/nr-reporting-summary-flat.pdf

Life sciences study design

All studies must disclose on these points even when the disclosure is negative.

Sample size	This study enrolled 1795 patients with neurodegenerative dementia, including 1592 patients with Alzheimer's disease (AD), 110 patients with frontotemporal dementia (FTD), 93 patients with dementia with Lewy bodies (DLB). This is a cross-sectional study, it is mainly included based on the prevalence of these three disease in clinical practice. AD is the most common neurodegenerative dementia, FTD is the second most common cause of neurodegenerative dementia after AD in patients younger than 65 years and DLB has been reported as being the second most common dementia subtype in older people following AD.
Data exclusions	No data was excluded from the analysis.
Replication	All results are repeatable. First, we sequenced DNA of 1795 patients through targeted genes sequencing, and then verified all variants in this study by Sanger sequencing.
Randomization	This is not relevant to this study, because in this study we did not include control subjects, but refer to the mutation frequency of related gene mutations in different population in the database as a control.
Blinding	In the process of data collection and analysis, the blind method is strictly followed.

Reporting for specific materials, systems and methods

We require information from authors about some types of materials, experimental systems and methods used in many studies. Here, indicate whether each material, system or method listed is relevant to your study. If you are not sure if a list item applies to your research, read the appropriate section before selecting a response.

Materials & experimental systems

n/a	Involved in the study	
☒	☐	Antibodies
☒	☐	Eukaryotic cell lines
☒	☐	Palaeontology and archaeology
☒	☐	Animals and other organisms
☐	☒	Human research participants
☒	☐	Clinical data
☒	☐	Dual use research of concern

Methods

n/a	Involved in the study	
☒	☐	ChIP-seq
☒	☐	Flow cytometry
☒	☐	MRI-based neuroimaging

Human research participants

Policy information about studies involving human research participants

Population characteristics	age at onset, age at diagnosis, gender, family history of dementia, disease duration, MMSE scores, APOE genotypes.
Recruitment	All patients were recruited from the Cognitive Impairment Disease Multicenter Database and Collaborative Network in China (CI-MDCNC).
Ethics oversight	This study was approved by the Ethics Committee of Xiangya Hospital, Central South University, China.

Note that full information on the approval of the study protocol must also be provided in the manuscript.
